# Parenteral nutrition support for patients with pancreatic cancer. Results of a phase II study

**DOI:** 10.1186/1471-2407-10-86

**Published:** 2010-03-09

**Authors:** Uwe Pelzer, Dirk Arnold, Mehmet Goevercin, Jens Stieler, Bernd Doerken, Hanno Riess, Helmut Oettle

**Affiliations:** 1 Universitätsmedizin Berlin, Charité Centrum für Tumormedizin, CONKO - Study Group, Augustenburger Platz 1, Berlin, Germany; 2 Universitätsklinikum Halle, Abteilung für Innere Medizin, Ernst-Gruber-Str. 40, Halle, Germany; 3 Evangelisches Geriatriezentrum Berlin GmbH, Reinickendorfer Straße 61, Berlin, Germany

## Abstract

**Background:**

Cachexia is a common problem in patients (pts) suffering from upper gastrointestinal cancer. In addition, most of these patients suffer from malabsorption and stenosis of the gastrointestinal tract due to their illness. Various methods of supplementary nutrition (enteral, parenteral) are practised. In patients with advanced pancreatic cancer (APC), phase angle, determined by bio-electrical impedance analysis (BIA), seems to be a survival predictor. The positive influence of BIA determinate predictors by additional nutrition is currently under discussion.

**Methods:**

To examine the impact of additional parenteral nutrition (APN) we assessed outpatients suffering from APC and progressive cachexia. The assessment based on the BIA method. Assessment parameters were phase angle, ECM/BCM index (ratio of extracellular mass to body cell mass), and BMI (body mass index). Patients suffering from progressive weight loss in spite of additional enteral nutritional support were eligible for the study.

**Results:**

Median treatment duration in 32 pts was 18 [8-35] weeks. Response evaluation showed a benefit in 27 pts (84%) in at least one parameter. 14 pts (43.7%) improved or stabilised in all three parameters. The median ECM/BCM index was 1.7 [1.11-3.14] at start of APN and improved down to 1.5 [1.12-3.36] during therapy. The median BMI increased from 19.7 [14.4-25.9] to 20.5 [15.4-25.0]. The median phase angle improved by 10% from 3.6 [2.3-5.1] to 3.9 [2.2-5.1].

**Conclusions:**

We demonstrated the positive impact of APN on the assessed parameters, first of all the phase angle, and we observed at least a temporary benefit or stabilisation of the nutritional status in the majority of the investigated patients. Based on these findings we are currently investigating the impact of APN on survival in a larger patient cohort.

**Trial registration:**

ClinicalTrials.gov Identifier: NCT00919659

## Background

Pancreatic adenocarcinoma is a highly aggressive cancer type being nearly chemoresistant, characterised by early local spread, extensive invasion and precocious metastasis. It is associated with marked cachexia. In spite of intensive experimental research in the last decade, the five year survival rate is still less than 5% [1,2]. Besides rapid disease progression, patients suffer from merged collateral symptoms including abdominal pain, nausea, emesis, inability for natural nutrition intake, taste abnormalities, early satiety, fatigue, stenosis, malabsorption and maldigestion. These additional symptoms contribute substantially to degradation in the performance status and quality of life [3-5]. The combination of these symptoms, usually called "cancer anorexia-cachexia syndrome", is considered an independent predictor of mortality and poor therapeutic response [6]. Nearly 50% of the patients with gastrointestinal malignancies suffer from this debilitating disease, whose most important phenotypic feature is muscle wasting and functional impairment caused by protein degradation combined with reduced protein synthesis [7]. Patients affected with advanced pancreatic cancer have the highest incidence of cancer cachexia, amounting to nearly 80 per cent of pts. at the time of diagnosis [6]. Nutritional support has often been practised in comprehensive cancer therapy. Various configurations of support have been used to improve or stabilise patient performance status, prognosis and response to therapy and also to reduce the complications of treatment.

Common evaluation parameters of the nutritional status (e.g. weight change, mid arm muscle circumference, triceps skin fold thickness) or laboratory measurements are unstable in the clinical setting of cancer patients. Some of the serum parameters (e.g. serum albumin, transferrin) are likely to be influenced by many non nutritional factors [8]. A more objective assessment is provided by the BIA method. BIA measurements and particularly the parameter phase angle, have been proven suitable for evaluating the nutritional status in APC patients. Phase angle estimation may be used for survival prediction [9].

The method is based on the electrical characteristics of the human body. The key characteristic is the almost complete conduction of a fixed, low voltage, high frequency alternating current through the fluid compartment of the fat-free mass in the human body [10]. The body component resistance (R) and capacitance (Xc) are measured by estimating a voltage discrepancy in the applied current. The resistive effect (Xc) at tissue interfaces and cell membranes generates a phase shift. The shift is quantified geometrically as the angular transformation of the capacitance to resistance ratio, referred to as phase angle [11]. Phase angle reflects the relative contributions of fluid (R) and cellular membranes (Xc) and is positively associated with capacitance and negatively with resistance [11]. It characterises the distribution of water between the extracellular and the intracellular spaces, which is one of the most sensitive indicators of malnutrition [12]. The ECM/BCM index describes the nutritional status in a similar way [13]. BCM is the whole cell mass responsible for metabolism; ECM includes the connective tissues such as collagen, elastin, skin, chords, bones as well as interstitial water (ascites, pleural effusion etc.). In healthy individuals, the BCM is always distinctly higher than the ECM, so the index is < 1 [13]. A rising ECM/BCM index is an early warning sign of a worsening nutritional status. However, the index is also influenced by over-hydration or dehydration of the body.

In the current study, we investigated the impact of additional parenteral nutrition on the nutritional status in APC patients by using BIA parameters like phase angle, ECM/BCM index and BMI.

## Methods

Ambulant patients with stage IV inoperable pancreatic cancer and reduced nutritional status were evaluated between January 2002 and January 2004 (Table 1). All patients gave their informed consent to the evaluation, the trial was conducted in compliance with the Helsinki Declaration, was approved by the local ethics committee and registered in accordance with the International Committee of Medical Journal Editors (NCT00919659). First a dietician assessed the baseline nutritional status in all patients. The clinical check up was arranged by the supervising physician. Candidates for APN were patients with weight losses over 5% in the previous four weeks or BMI below 19 in spite of additional enteral caloric support (carbohydrate suspensions 200-400 ml, 1.5 kcal/ml) combined with drug support (antiemetic, corticosteroid, prokinetics, gestagen, cannabinoids). Almost all patients suffered from gastrointestinal stenosis, gastro-paresis and loss of appetite. The potential changes in the nutritional status during our intervention were assessed by BIA. The BIA measurements were performed according to the common operating manual guidelines exemplified in the paperwork by Gupta et al. [9]. The fundamental design parameters were as follows: patients in horizontal supine position on an examination table, extremities apart and not touching each other or the torso; the four surface standard electrode (tetrapolar) technique was used in such a way that two electrodes were placed on the right hand and the remaining two electrodes on the right leg. Resistance, capacitance and phase angle were directly measured (optimal calibration at 50 kHz, 800 μA). ECM, BCM and BMI were calculated. Statistic calculations were done by using SPSS 11.5 (SPSS Inc., Chicago, IL, USA).

**Table 1:** Patient characteristics at start of APN

Character		Range
Screened patients	65	
Recruited patients	32	
Female	14	
Male	18	
Age	62 years	[47-75]
Histology	pancreatic adenocarcinoma	
Metastatic disease	32	
Median BMI	19.7 kg/m^2^	[14.4 to 25.0]
Median Phase angle	3.6°	[2.3 to 5.1]
Median ECM/BCM index	1.7	[1.1 to 3.1]

APN was arranged on an overnight home treatment basis and consisted of caloric intake of about 25 kcal/kg daily on five out of seven days (amino-acids 1.2 to 1.5 g/kg, lipids at least 35% of the whole energy support, additional vitamins or electrolyte if indicated, no additional glutamine or ω-3 fatty acid). Patient height and body weight were measured using a calibrated scale to calculate the individual BMI. Response was evaluated according to Table 2.

**Table 2:** Nutritional status - study assessment rules

Parameter	Improvement	Stabilisation	Impairment
BMI	> + 5%	+/- 5%	> - 5%
ECM/BCM index	> - 5%	+/- 5%	> + 5%
Phase angle	> + 5%	+/- 5%	> - 5%

## Results

**Figure 1:**
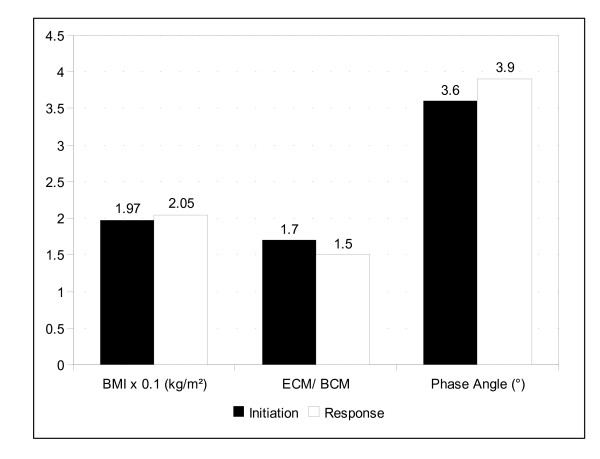
**Median best response of study cohort in terms of BMI, ECM/BCM index and phase angle**.

Sixty five ambulant pts with histologically proven APC had been screened between January 2002 and January 2004 in our university outpatient clinic in Berlin/Germany. 32 of them suffered from marked progressive cachexia and were willing to receive APN. Median treatment duration was 18 (8-35) weeks. The nutritional status was evaluated by BIA every 4 to 6 (2-8) weeks. Figure 1 represents the median response. The median BMI at start of APN was 19.7 (14.4-25.9) and increased to 20.5 (15.4-25.0) during APN therapy. The median ECM/BCM index at start of APN was 1.7 (1.11-3.14) and decreased to 1.5 (1.12-3.36). The main parameter, phase angle, increased by 10%, from 3.6 (2.3-5.1) to 3.9 (2.2-5.1). Nearly half the patients (15/32) had a temporarily improved phase angle, and in 13/32 pts. we observed a stabilisation of this parameter. Only 13% (4/32) of pts. showed a decrease in phase angle in spite of APN.

**Figure 2:**
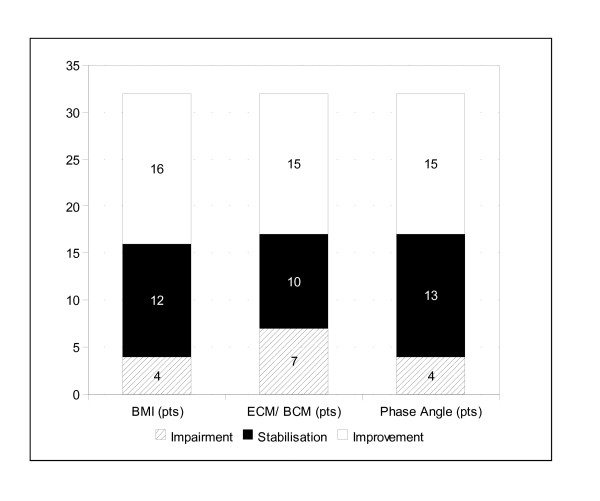
**Number of individual responders in terms of BMI, ECM/BCM index or phase angle**.

Figure 2 typifies the specific response. Improvement in at least one parameter was seen in 27 of the 32 pts (84%). In 15 pts.(46%) we observed an improvement in two main parameters of nutritional performance. 9 pts. (28%) improved in all three parameters (BMI, phase angle, ECM/BCM index). 12 pts. (38%) stabilised in two of the main parameters, 5 pts. (16%) in all three parameters. In 5 pts (16%) APN was without any positive effect, 3 of them did even degrade in all three parameters while receiving APN. By longer treatment, beyond the achieved response, we do not obtain higher response. All patients were still alive at the end of the intervention. No severe side effects (e.g. over-hydration, electrolyte disturbances, venous port infection) were observed.

## Discussion

Only few data are available to shed light on the prognostic impact of phase angle on overall survival in pancreatic cancer [9]. The goal of our study was phase angle improvement via APN. The majority of the patients supported by APN showed an improved nutritional status, which was verified by changes in the target parameters.

Phase angle improved from 3.6 to 3.9, the ECM/BCM index dropped from 1.7 down to 1.5, and the BMI increased slightly from 19.7 to 20.5. However, overall normalisation of the BIA parameters was not achieved by APN.

Our study design did not allow us to draw conclusions as to the clinical relevance (eg.: overall survival, quality of life) of the findings. A randomised study design with a control group (no APN support) would probably have yielded more powerful data. But for ethical concerns, conduction of such a study is absolutely out of the question.

Nevertheless we demonstrated the positive effect of the study intervention on the nutritional status of the patients. The pre-existing data support the direct correlation between overall survival and phase angle in patients with APC [9]. Beside BMI or specific weight loss, the ECM/BCM index proved to be a useful tool for nutritional assessment. A decreasing ECM/BCM index resulted in a recovery in patients with malnutrition due to gastrointestinal non-malignant diseases [14].

If baseline and changes in the nutritional status are determined by BIA parameters alone, limitations of the BIA technique should be taken into account. A potential limitation is its reliance on regression models that are derived from a limited number of human subjects. These models fail to work properly in patients who are different from the original patient sample [15,16]. Variability in the direct bio-impedance measures (resistance, capacitance and phase angle) is likely due to age, gender, and body mass characteristics of the study population.

Other reported limitations are hydration status and major disturbances of water distribution, body position during the procedure, ambient air and skin temperatures, recent physical activity, conductance of the examining table, and food consumption [15,17]. Not all of these factors could be controlled in this trial. Yet signs of over-hydration were under control and all our patients were found free from visible oedema or ascites. Body position was controlled at examination, extreme physical activity was anyway most unlikely in these patients. Air temperature was within the controlled range in the outpatient department.

Difficulties in the process of analysis were caused by the individually tailored chemotherapy, supportive drugs, patient opinion, course of disease and complicated assessment of remaining enteral nutrition. Due to the small size of the study we were not able to exclude these influencing variables or arrange them in subgroups. Patients were treated with APN until they or their physicians did not see any further benefit from it.

## Conclusions

In summary, our present study has demonstrated the positive impact of APN on the nutritional status of patients with APC. Proceeding from these results we have started the next study phase with a larger patient cohort to correlate the level of nutritional improvement with overall survival and quality of life.

The decision if APN was indicated or not was taken in accordance with the current ESPEN (European Society for Clinical Nutrition and Metabolism) guidelines, which are congruent with our appraisal [18]. Home parenteral nutrition may be recommended for hypophagic/(sub)obstructed cancer patients with acceptable performance status if they are expected to die from starvation prior to cancer spread.

## Competing interests

The authors declare that they have no competing interest.

The grant from Fresenius Kabi Deutschland GmbH was used for employing a dietician.

## Authors' contributions

HO and UP were investigators. UP drafted the manuscript. UP, MG, JS, DA recruited patients into the study. BD, HR, HO provided the trial facility. All authors read and approved the final manuscript.

## Pre-publication history

The pre-publication history for this paper can be accessed here:

http://www.biomedcentral.com/1471-2407/10/86/prepub
